# Supplementary material for: A randomized stepped wedge trial of an intensive combination approach to roll back the HIV epidemic in Nigerian adolescents: iCARE Nigeria treatment support protocol
Source: PLoS One. 2023 Jul 7;18(7):e0274031. doi: 10.1371/journal.pone.0274031 (PMC10328338; doi:10.1371/journal.pone.0274031)
Supplement: S1 File — (DOCX) [file pone.0274031.s001.docx]

| **Version Information** | **Summary of Revisions Made** | **Rationale** |
| --- | --- | --- |
| Version 2.0 | 1. The study timelines associated with Study 1 and Study 2 were corrected to be consistent with the Study Schema (i.e., Time 1=0-24 weeks)  2. In Study 2, the inclusion criterion for language ability was revised to include two additional languages: Hausa and Pidgin English | 1. The study timelines are now consistent with the Study Schema.  2. In Study 2, the inclusion criterion for language ability now includes the primary languages reflected in the study population. |
| Version 3.0 | 1. We have removed the enrollment cap of only 35% suppressed participants at clinic sites.  2. The inclusion criteria were revised to allow participation of non-emancipated 15-year-old youth, with parental consent.  3. Exclusion criteria were revised to exclude non-emancipated 15-year-old youth who do not have parental consent.  4. We have removed a reference to DNA extraction in the description of DBS procedures.  5. We have added a requirement for parental consent to the consent procedures.  6.We have revised the consent process for participants who are assented at enrollment, to consent them as the earliest opportunity after they reach the age of consent. | 1. The proportion of suppressed patients varies by clinic site and thus there is no one proportion suppressed that is representative for all clinics.  2. We have broadened the inclusion criteria for youth who have reached an age of transition to adult care at age 15, but who are not emancipated.  3. Exclusion criteria were revised to make it clear that non-emancipated 15-year-old youth who do not have parental consent are excluded.  4. The reference to DNA extraction in the description of DBS procedures was an error.  5. The inclusion of non-emancipated 15-year old youth in clinical research in Nigeria requires parent consent.  6. We will consent patients who were assented at enrollment to promote their autonomy to consent for research participation. |
| Version 4.0 | 1. We have updated the description of sample size in study 2 to N=560.  2. In study 1, we have revised the protocol to include abstraction of HIV testing, behavioral and demographic data from sexual partners of eligible individuals tested via iCARE, regardless of sex and age (see updated Consent Statement, Version 2, March 11, 2022).  3. We have updated reporting of SAEs such that only SAEs that are unanticipated, and related or possibly related to the study will be reported to the IRB and the DSMB within 7 working days of the study team becoming aware of its occurrence. All SAEs (regardless of whether they are unanticipated or related or possibly related to the study) will be reported to the Northwestern team and the NIH Program Officer within 3 days of occurrence.  4. With Study PI approval, a remote verbal consent addendum was added to allow HIV treatment arm participants who have moved or are otherwise not available for an in-person follow up study visit at their study site to complete the viral load testing and questionnaires from their current location (see Verbal Consent Addendum, Version 1, March 11, 2022). | 1. The study sample size has been updated for specificity and to align with the Study Schema  2. HIV testing data from sexual partners of eligible cases provides an opportunity to collect additional information on HIV related risk for the target population.  3. The SAE reporting criteria were revised to be consistent with reporting requirements at site IRBs, which only require reporting of SAEs that meet these criteria and to respond to the DSMB preference for reporting.  4. Remote completion of a follow-up study visit will be allowed on rare occasions to limit loss-to-follow-up for the primary endpoint (viral load). |

**Intensive Combination Approach to Rollback the Epidemic in Nigerian Adolescents: UH3 Phase**

**Protocol Number: iCARE Nigeria UH3**

**National Clinical Trial (NCT) Identified Number: Pending**

**Nigerian Clinical Trial Registry (NCTR): Pending**

**Principal Investigators (PI):**

**Local PIs: Olayinka Omigbodun, MBBS, MPH, FAS University of Ibadan, University College Hospital, Infectious Disease Institute (IDI/CoMUI)**

**Alani Sulaimon Akanmu, MBBS, Lagos University Teaching Hospital (LUTH)**

**Agatha David, MBBS, MPH, FMCPaed, Nigerian Institute of Medical Research (NIMR)**

**Akinsegun Akinbami , MBBS Lagos State University Teaching Hospital (LSUTH)**

**Oche O. Agbaji** **MBBS, MPH, FMCP, MACP, Jos University Teaching Hospital (JUTH)**

**Folashade Adekanmbi, Olabisi Onabanjo University Teaching Hospital, Sagamu (OOUTH/Sagamu)**

**Study PIs/Institutions: Babafemi Taiwo, MBBS, FAS Northwestern University**

**Robert Garofalo, MD, MPH, Lurie Children’s Hospital**

**Sponsor: NIH/NICHD**

**Grant Title: Prevention and Treatment through a Comprehensive Care Continuum for HIV-affected Adolescents in Resource Constrained Settings (PATC3H) (UG3/UH3)**

**Grant Number*: UH3HD096920**

**Funded by: Eunice Kennedy Shriver National Institute of Child Health and Human Development**

**Version Number: 4.0**

**011 March 2022**

**Summary of Changes from Previous Version:**

| **Affected Section(s)** | **Summary of Revisions Made** | **Rationale** |
| --- | --- | --- |
| **4.1**  **5.1** | 1. The study timelines associated with Study 1 and Study 2 were corrected to be consistent with the Study Schema (i.e., Time 1=0-24 weeks)  2. In Study 2, the inclusion criterion for language ability was revised to include two additional languages: Hausa and Pidgin English | 1. The study timelines are now consistent with the Study Schema.  2. In Study 2, the inclusion criterion for language ability now includes the primary languages reflected in the study population. |
| **4.1, 5.1, 5.5**  **5.1**  **5.2**  **8.1**  **10.1.1.1,**  **10.1.1.2** | 1. We have removed the enrollment cap of only 35% suppressed participants at clinic sites.  2. The inclusion criteria were revised to allow participation of non-emancipated 15-year-old youth, with parental consent.  3. Exclusion criteria were revised to exclude non-emancipated 15-year-old youth who do not have parental consent.  4. We have removed a reference to DNA extraction in the description of DBS procedures.  5. We have added a requirement for parental consent to the consent procedures.  6.We have revised the consent process for participants who are assented at enrollment, to consent them as the earliest opportunity after they reach the age of consent | 1. The proportion of suppressed patients varies by clinic site and thus there is no one proportion suppressed that is representative for all clinics.  2. We have broadened the inclusion criteria to include youth who have reached an age of transition to adult care at age 15, but who are not emancipated.  3. Exclusion criteria were revised to make it clear that non-emancipated 15-year-old youth who do not have parental consent are excluded.  4. The reference to DNA extraction in the description of DBS procedures was an error.  5. The inclusion of non-emancipated 15-year old youth in clinical research in Nigeria requires parent consent.  6. We will consent patients who were assented at enrollment to promote their autonomy to consent for research participation. |
| **4.1**  **5.1**  **8.2.5** | 1. We have updated the description of sample size in study 2 to N=560.  2. In study 1, we have revised the protocol to include abstraction of HIV testing, behavioral and demographic data from sexual partners of eligible individuals tested via iCARE, regardless of sex and age (see updated Consent Statement, Version 2, March 11, 2022).  3. We have updated reporting of SAEs such that only SAEs that are unanticipated, and related or possibly related to the study will be reported to the IRB and the DSMB within 7 working days of the study team becoming aware of its occurrence. All SAEs (regardless of whether they are unanticipated or related or possibly related to the study) will be reported to the Northwestern team and the NIH Program Officer within 3 days of occurrence. | 1. The study sample size has been updated for specificity and to align with the Study Schema  2. HIV testing data from sexual partners of eligible cases provides an opportunity to collect additional information on HIV related risk for the target population.  3. The SAE reporting criteria were revised to be consistent with reporting requirements at site IRBs, which only require reporting of SAEs that meet these criteria and to respond to the DSMB preference for reporting. |

Table of Contents

[STATEMENT OF COMPLIANCE 1](#_Toc4165618)

[INVESTIGATOR’S SIGNATURE 2](#_Toc4165619)

[1 PROTOCOL SUMMARY 3](#_Toc4165620)

[1.1 Synopsis 3](#_Toc4165621)

[1.2 Schema 5](#_Toc4165622)

[1.3 Schedule of Activities 6](#_Toc4165623)

[2 INTRODUCTION 8](#_Toc4165624)

[2.1 Study Rationale 8](#_Toc4165625)

[2.2 Background 8](#_Toc4165626)

[2.3 Risk/Benefit Assessment 9](#_Toc4165627)

[2.3.1 Known Potential Risks 9](#_Toc4165628)

[2.3.2 Known Potential Benefits 9](#_Toc4165629)

[2.3.3 Assessment of Potential Risks and Benefits 9](#_Toc4165630)

[3 OBJECTIVES AND ENDPOINTS 10](#_Toc4165631)

[4 STUDY DESIGN 12](#_Toc4165632)

[4.1 Overall Design 12](#_Toc4165633)

[4.2 Scientific Rationale for Study Design 15](#_Toc4165634)

[4.3 Justification for Intervention 16](#_Toc4165635)

[4.4 End-of-Study Definition 16](#_Toc4165636)

[5 STUDY POPULATION 17](#_Toc4165637)

[5.1 Inclusion Criteria 17](#_Toc4165638)

[5.2 Exclusion Criteria 17](#_Toc4165639)

[5.3 Lifestyle Considerations 17](#_Toc4165640)

[5.4 Screen Failures 18](#_Toc4165641)

[**5.5** Strategies for Recruitment and Retention 18](#_Toc4165642)

[6 STUDY INTERVENTION(S) OR EXPERIMENTAL MANIPULATION(S) 18](#_Toc4165643)

[6.1 Study Intervention(s) or Experimental Manipulation(s) Administration 18](#_Toc4165644)

[6.2 Fidelity 20](#_Toc4165645)

[6.2.1 Interventionist Training and Tracking 20](#_Toc4165646)

[6.3 Measures to Minimize Bias: Randomization and Blinding 20](#_Toc4165647)

[6.4 Study Intervention/Experimental Manipulation Adherence 20](#_Toc4165648)

[6.5 Concomitant Therapy 21](#_Toc4165649)

[7 STUDY INTERVENTION/EXPERIMENTAL MANIPULATION DISCONTINUATION AND PARTICIPANT DISCONTINUATION/WITHDRAWAL 21](#_Toc4165650)

[7.1 Discontinuation of Study Intervention/Experimental Manipulation 21](#_Toc4165651)

[7.2 Participant Discontinuation/Withdrawal from the Study 21](#_Toc4165652)

[7.3 Lost to Follow-Up 21](#_Toc4165653)

[8 STUDY ASSESSMENTS AND PROCEDURES 21](#_Toc4165654)

[8.1 Endpoint and Other Non-Safety Assessments 21](#_Toc4165655)

[8.2 Safety Assessments 23](#_Toc4165656)

[Adverse Events and Serious Adverse Events 24](#_Toc4165657)

[8.2.1 Definition of ADVERSE EVENTS AND Serious Adverse Events 24](#_Toc4165658)

[8.2.2 Classification of an Adverse Event 24](#_Toc4165659)

[8.2.3 Time Period and Frequency for Event Assessment and Follow-Up 24](#_Toc4165660)

[8.2.4 Adverse Event Reporting 24](#_Toc4165661)

[8.2.5 Serious Adverse Event Reporting 24](#_Toc4165662)

[8.2.6 Reporting Events to Participants 25](#_Toc4165663)

[8.2.7 Events of Special Interest 25](#_Toc4165664)

[8.2.8 Reporting of Pregnancy 25](#_Toc4165665)

[8.3 Unanticipated Problems 25](#_Toc4165666)

[8.3.1 Definition of Unanticipated Problems 25](#_Toc4165667)

[8.3.2 Unanticipated Problems Reporting 26](#_Toc4165668)

[8.3.3 Reporting Unanticipated Problems to Participants 26](#_Toc4165669)

[9 STATISTICAL CONSIDERATIONS 26](#_Toc4165670)

[9.1 Statistical Hypotheses 26](#_Toc4165671)

[9.2 Sample Size Determination 27](#_Toc4165672)

[9.3 Populations for Analyses 27](#_Toc4165673)

[9.4 Statistical Analyses 27](#_Toc4165674)

[9.4.1 General Approach 27](#_Toc4165675)

[9.4.2 Analysis of the Primary Endpoint(s) 27](#_Toc4165676)

[9.4.3 Analysis of the Secondary Endpoint(s) 28](#_Toc4165677)

[9.4.4 Safety Analyses 28](#_Toc4165678)

[9.4.5 Baseline Descriptive Statistics 28](#_Toc4165679)

[9.4.6 Planned Interim Analyses 28](#_Toc4165680)

[9.4.7 Sub-Group Analyses 29](#_Toc4165681)

[9.4.8 Tabulation of Individual Participant Data 29](#_Toc4165682)

[9.4.9 Exploratory Analyses 29](#_Toc4165683)

[10 SUPPORTING DOCUMENTATION AND OPERATIONAL CONSIDERATIONS 29](#_Toc4165684)

[10.1 Regulatory, Ethical, and Study Oversight Considerations 29](#_Toc4165685)

[10.1.1 Informed Consent Process 29](#_Toc4165686)

[10.1.2 Study Discontinuation and Closure 30](#_Toc4165687)

[10.1.3 Confidentiality and Privacy 31](#_Toc4165688)

[10.1.4 Future Use of Stored Specimens and Data 31](#_Toc4165689)

[10.1.5 Key Roles and Study Governance 31](#_Toc4165690)

[10.1.6 Safety Oversight 31](#_Toc4165691)

[10.1.7 Clinical Monitoring 32](#_Toc4165692)

[10.1.8 Quality Assurance and Quality Control 32](#_Toc4165693)

[10.1.9 Data Handling and Record Keeping 32](#_Toc4165694)

[10.1.10 Protocol Deviations 33](#_Toc4165695)

[10.1.11 Publication and Data Sharing Policy 33](#_Toc4165696)

[10.1.12 Conflict of Interest Policy 33](#_Toc4165697)

[10.2 Additional Considerations 33](#_Toc4165698)

[10.3 Abbreviations and Special Terms 33](#_Toc4165699)

[10.4 Protocol Amendment History 34](#_Toc4165700)

[11 REFERENCES 35](#_Toc4165701)

# STATEMENT OF COMPLIANCE

The trial will be carried out in accordance with International Council on Harmonisation Good Clinical Practice (ICH GCP) and the following:

- United States (US) Code of Federal Regulations (CFR) applicable to clinical studies (45 CFR Part 46, 21 CFR Part 50, 21 CFR Part 56, 21 CFR Part 312, and/or 21 CFR Part 812).
- The Nigerian National Code for Health Research Ethics (www.nhrec.net)

National Institutes of Health (NIH)-funded investigators and clinical trial site staff who are responsible for the conduct, management, or oversight of NIH-funded clinical trials have completed Human Subjects Protection and ICH GCP Training.

The protocol, informed consent form(s), recruitment materials, and all participant materials will be submitted to the IRB for review and approval. Approval of both the protocol and the consent form(s) must be obtained before any participant is consented. Any amendment to the protocol will require review and approval by the IRB before the changes are implemented to the study. All changes to the consent form(s) will be IRB approved; a determination will be made regarding whether a new consent needs to be obtained from participants who provided consent, using a previously approved consent form.

# INVESTIGATOR’S SIGNATURE

The signature below constitutes the approval of this protocol and provides the necessary assurances that this study will be conducted according to all stipulations of the protocol, including all statements regarding confidentiality, and according to local legal and regulatory requirements and applicable US federal regulations and ICH guidelines.

Principal Investigator or Clinical Site Investigator:

| Signed: |  | Date: |  |
| --- | --- | --- | --- |
|  | Name^*^: | | |
|  | Title^*^: | | |

Investigator Contact Information
Affiliation^*^:

Address:

Telephone:

Email:

| Signed: |  | Date: |  |
| --- | --- | --- | --- |
|  | Name: | | |
|  | Title: | | |
|  | Affiliation: | | |

# PROTOCOL SUMMARY

## Synopsis

| **Title:** | | Intensive Combination Approach to Rollback the Epidemic in Nigerian Adolescents (iCARE Nigeria): UH3 Phase | |
| --- | --- | --- | --- |
| **Grant Number:** | | UH3HD096920 | |
| **Study Description:** | | Two combination interventions, each with mHealth and Peer Navigation components, will be evaluated in a randomized, stepped wedge trial among youth in Ibadan, Lagos, Sagamu, and Jos, Nigeria. Study findings will demonstrate whether or not the combination interventions, which were found to be efficacious in our prior UG3 trial, will remain efficacious if scaled as proposed in this UH3 trial, across multiple sites. | |
| **Objectives:** | | The primary objective of the HIV Testing Intervention is to determine the effect of Social Media Engagement + Peer Navigation on HIV seroprevalence among young men across multiple testing sites*.* A secondary objective is to determine the effect of the intervention on linkage of newly diagnosed young men to a clinic for antiretroviral therapy (ART).  The primary objective of the HIV Treatment Intervention is to determine the efficacy of the combination of SMS + Peer Navigation to improve viral suppression among youth with HIV receiving ART across multiple sites. Secondary objectives are to determine the effect of the intervention on medication adherence and retention in care. A tertiary objective is to test ART adherence and retention in care as mediators of the intervention effect as well as age, gender, and mode of transmission as potential moderators of the intervention effect across multiple sites.  An exploratory objective is to identify and describe the barriers and facilitators of iCARE intervention readiness and implementation (both interventions) across multiple sites and to describe intervention costs. | |
| **Endpoints:** | | The primary endpoint for the HIV Testing Intervention is the change in HIV seroprevalence during the intervention period in comparison to the control period.  The primary endpoint for the HIV Treatment Intervention is rate of viral suppression, defined as viral load <200 copies/mL, during intervention period compared to the control period. | |
| **Study Population:** | |  | |
|  | | The population for the HIV Testing Intervention is the total number of HIV tests completed, and resulting seroprevalence, among youth in/around Ibadan, Lagos, Sagamu and Jos.  The study population for the HIV Treatment Intervention are youth living with HIV and receiving antiretroviral therapy (ART) at IDI/CoMUI, LUTH, NIMR, LASUTH, OOUTH Sagamu and JUTH, or their satellite clinics. | |
| **Phase or Stage:** | | This is the UH3, second phase of the iCARE Nigeria study | |
| **Description of Sites/Facilities Enrolling Participants:** | | HIV Programs at IDI/CoMUI, LUTH, NIMR, LASUTH, OOUTH Sagamu and JUTH are supported by the APIN Public Health Initiatives to provide ART to persons living with HIV (PLWH). | |
| **Description of Study Intervention/Experimental Manipulation:** | | The combination intervention in each of the studies includes peer navigation and mHealth components | |
| **Study Duration:** | | The study duration is 96 weeks (from start of data collection to end of data collection) in the stepped wedge design. Additionally, abstraction of HIV Testing surveillance data and HIV Treatment medical data will continue through 120 weeks, respectively, in order to explore the durability of the intervention effects post-intervention. | |

## Schema


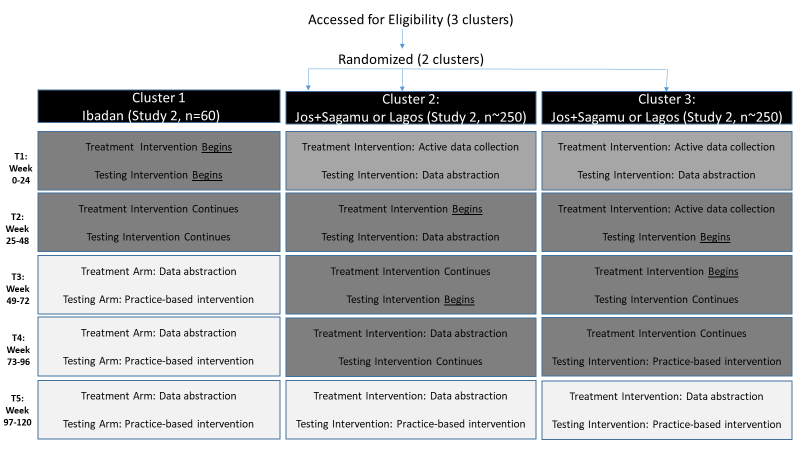


| Pre-Intervention Phase |  |
| --- | --- |
| Intervention Phase |  |
| Post-Intervention Phase |  |

## Schedule of Activities

**Study 1 (HIV Testing Intervention and Linkage to Care)**

|  | Pre-intervention | Intervention Phase | Post-intervention |
| --- | --- | --- | --- |
| Activities | - Collect HIV testing seroprevalence data from each site for young men ages 15-24 for the period preceding intervention - Collect questionnaire and qualitative data (focus groups, key informant interviews) on intervention readiness from study staff and CBO representatives | - Social Media Engagement - Community Outreach and Peer Navigation including HIV Testing - Collect measures of intervention implementation from study staff and CBO representatives - Collect intervention cost data from site administrative records and through interviews with stakeholders | - Collect questionnaire and qualitative data on intervention implementation from study staff and CBO representatives |

**Study 2 (HIV Treatment Intervention)**

|  | | Pre-intervention (Control Sites) | Baseline (Intervention Phase) Intervention | Week 24- Intervention Phase | Week 48 – Intervention Phase | Post-intervention |
| --- | --- | --- | --- | --- | --- | --- |
| Informed consent | | X | X |  |  |  |
| Blood draw for viral load | | X | X | X | X | X^a^ |
| Blood draw for CD4 count | | X^b^ | X^b^ |  |  |  |
| Blood draw for future studies (stored; virologic resistance testing and other studies) | | X | X | X | X |  |
| Adherence Assessment: Dried Blood Spot (DBS), Pharmacy Pick-up Records Abstraction, Self-Report | | X | X | X | X | X^c^ |
| Computer Assisted Personal Interviewing Questionnaires ^d^ | | X | X | X | X |  |
| Implementation Assessment ^e^ | | X | X | X | X | X |
| Cost Assessment ^f^ | | X | X | X | X | X |
| **SMS + Peer Navigation** | |  | Baseline through week 48 | | |  |
| SMS Text Initiation | |  | X |  |  |  |
| Peer Navigation Enrollment | |  | X |  |  |  |
|  | |  |  |  |  |  |
|  | ^a^ Per medical chart abstraction of standard of care viral load quantification  ^b^ CD4 quantification will be performed only at the first study visit (either pre-intervention in Jos, Lagos, Sagamu), or baseline (in Ibadan)  ^c^ Medical records abstraction only (no self-report, no DBS)  ^d^ Computer assisted personal interviewing (CAPI): Demographic Characteristics, HIV Treatment Knowledge, HIV Medication Self-Efficacy, HIV Stigma, Substance Use, Depressive Symptoms, Intervention Satisfaction and Acceptability  ^e^ Includes Questionnaires, Key Informant Interviews, and Focus groups with study and clinic staff, CBO representatives  ^f^ Includes abstraction of cost data from site administrative records and through interviews with stakeholders | | | | |  |

# INTRODUCTION

## Study Rationale

There is a need for effective interventions to improve HIV diagnosis and treatment among high-risk youth in Nigeria.

## Background

Nigeria has more than 1.9 million people living with HIV/AIDS (PLWH; 1.5%),^1^ the second highest in the world, however, it has been largely neglected as a focus of public health efforts compared to other “hot spots” in sub-Saharan Africa.^2^ In 2018, Nigeria had 130,000 new HIV infections and 53,000 AIDS related deaths.^1^ Progress towards UNAIDS 95-95-95 goals in Nigeria is slow, with an estimated 67% of PLWH aware of their status, 55% on antiretroviral therapy (ART), and 42% virally suppressed.^3^

Nigeria has a mixed epidemic; meaning that risk of acquiring of HIV as well as access to HIV-testing and treatment services are not evenly distributed across demographic groups. Specifically, with regard to availability and uptake of HIV testing - a critical strategy in helping Nigeria achieve the UNAIDS 95-95-95 goals – youth and key populations such as men who have sex with men (MSM) are among those with disproportionate impacts.

Since 2016, guidelines in Nigeria, Africa’s most populous country, have recommended antiretroviral treatment regardless of CD4 cell count for all people with HIV.^4^ Effective ART suppresses HIV-1 replication, which curbs AIDS-related morbidity and mortality, limits viral transmission, and is essential to end the epidemic.^5-7^ Consistent with the global trend, youth (15-24 years of age) in Nigeria have not benefited from ART as much as adults.^8-11^ In 2017, fewer than half of the youth with HIV (YWH) at the Infectious Diseases Institute, University College Hospital, Ibadan, Nigeria (IDI/CoMUI) were virally suppressed, similar to the experience at other ART centers in the country (unpublished data). Evidence-based interventions are needed to optimize ART outcomes in YWH.^12^

Mobile phones are potentially powerful tools for ART intervention,^13,14^ and there were over 190 million active mobile phone subscriptions in Nigeria in April 2020.^15^ The widespread use and preference for text messaging among youth make it uniquely promising for youth-directed HIV prevention and ART interventions. In 2016, the United States (U.S.) Centers for Diseases Control (CDC) endorsed an innovative two-way text message reminder intervention (TXTXT) for promoting ART adherence in YWH.^16^ This endorsement was based on a study by Garofalo et al., in which 16-29-year-olds who received the TXTXT intervention were more than twice as likely to report >90% adherence to ART over the 6-month intervention period, and the improvements were sustained at 6-months post intervention.^17^ To date, TXTXT has not been evaluated outside the U.S.

Engagement of peers is another potential way to improve HIV testing and ART outcomes in youth, particularly adolescents (10-19 years of age) because peer influences on health and well-being are greatest during adolescence.^18-20^ Interventions that affect the attitudes, values, and behaviors of the peer group have the greatest impact during this stage of life.^21^ Indeed, peer navigation and support is an evidence-based approach that is recommended by the World Health Organization (WHO) to improve outcomes for youth across the HIV care continuum.^22^ Peer support ranks high among potential facilitators of ART adherence among YWH in sub-Saharan Africa,^23,24^ which harbored 78% of the global population of YWH in 2017.^25^

Studies of interventions to improve ART outcomes among 15-24-year-olds in sub-Saharan Africa have failed to demonstrate improvement in ART adherence or other outcomes, even when the same interventions were effective in older individuals.^26-28^ While these results reflect the unique struggles of youth, they may also indicate to the limitations of single interventions in addressing complex multi-dimensional determinants of ART success. There is evidence to suggest that combining multiple interventions may be more effective than single interventions,^29^ but this is not a consistent finding.^30^

The Intensive Combination Approach to Rollback the Epidemic in Nigerian Adolescents Study (iCARE Nigeria) is a two-phase trial with HIV testing and treatment intervention arms, each incorporating both mobile health technology (mHealth) and peer navigation components. iCARE Nigeria was adapted for Nigerian youth through broad input from local youth living with HIV and other stakeholders.^31^ Results from the UG3 phase of the iCARE study suggest both preliminary efficacy and feasibly of our combination intervention approach. In the HIV testing arm, during the 12-month intervention period, the iCARE intervention increased by >30% the number of HIV tests completed among the young men, ages 15-24 at IDI/CoMUI and increased the seropositivity rate among cases to 4.9%, compared to 1.7% in the 6-month period prior to the intervention. In the HIV treatment arm, the proportion of patients with a suppressed viral load (VL<200 copies/mL) increased by 71% over the 48-week intervention period, from 35% at baseline to 60% at 48 weeks, a statistically significant change (McNemar test, OR = 6.00, p = 0.013. Self-reported adherence ($\geq$90%) increased from baseline at both 24 weeks (63%, p = 0.008) and 48 weeks (68%, p = 0.031). Medication possession ratio (MPR) $\geq$90% increased numerically at both weeks 24 and 48 (85% and 80%, respectively), achieving statistical significance at 24 weeks only (p = 0.022). Retention in care at 48 weeks was 87.5%. Satisfaction as rated by individuals who completed HIV tests and participants in the HIV treatment intervention was over 90%.

## Risk/Benefit Assessment

### Known Potential Risks

The primary risk to participation in this study is breach of confidentiality (related to being MSM identified or to HIV status). However, the risk to the participant is no greater than that encountered in standard counseling or HIV treatment services. The risk associated with the collection of blood from a vein in the arm (for viral load quantification), include pain or discomfort at the site of collection, temporary bruising at that site and very rarely, the site of blood collection may become infected or need medical treatment.

### Known Potential Benefits

The purpose of this study is to determine whether the combination iCARE interventions, which were found to be efficacious across the HIV care continuum among youth ages 15-24 in the UG3 trial, will remain efficacious if scaled as proposed in this UH3 trial, across multiple sites in Nigeria. The results of the UG3 trial suggest potential beneficial effects of the intervention to improve rates of HIV testing and identification of seropositive cases, and to improve viral load suppression among youth living with HIV.

### Assessment of Potential Risks and Benefits

**Protection Against Risks**

For Study 1, the HIV Testing and Linkage to Care intervention, we will not enroll participants in research activities in order to reduce the potential risk of breach of confidentiality among YMSM given the criminalization of same-sex sexual behavior in Nigeria. In Study 2, The HIV Treatment Intervention, to protect the integrity of the youth’s data, staff will assign each individual a unique patient identification number (PID) at study enrollment, linked to their name and medical records number (for medical records abstraction). This code number will be used for all study data. Only staff who have been trained in human subjects protection will have access to these PIDs.

**Importance of the Knowledge to be Gained**

Possible risks (i.e. potential confidentiality breaches) are outweighed by the new knowledge gained from testing this intervention among the study population across multiple sites, a population at very high risk of poor HIV outcomes.

# OBJECTIVES AND ENDPOINTS

**Study 1: HIV Testing and Linkage to Care Intervention**

| OBJECTIVES | ENDPOINTS | JUSTIFICATION FOR ENDPOINTS | PUTATIVE MECHANISMS OF ACTION |
| --- | --- | --- | --- |
| Primary |  |  |  |
| To determine the effect of Social Media Engagement + Peer Navigation on HIV testing and seroprevalence among young men across multiple sites | HIV seroprevalence among the tested young men during the intervention period (calculated as the number of confirmed HIV cases divided by the total number of tests). Repeat testing in youth who initially test negative will be analyzed separately. | While the focus of this study is young men who have sex with men (YMSM), the social conditions in Nigeria make it difficult to collect reliable data from this population. Data from young men in general will be collected to overcome this obstacle. An increase in HIV testing among male youth regardless of their sexual orientation is also desirable to stem the HIV epidemic | The investigational intervention will alleviate barriers to HIV testing among young men, including YMSM |
| Secondary |  |  |  |
| To evaluate linkage of newly diagnosed young men to a clinic for ART | The percentage of young men who are linked to HIV care within 30 days of an HIV confirmatory test. This is defined as registering and completing a clinic visit where ART or opportunistic infection treatment is provided within 30 days of a confirmatory HIV test. | Ideally, all HIV diagnosed individuals would be linked to care; the public health goal is for 90% of HIV diagnosed individuals to be linked to care | Peer navigation will facilitate linkage to care |
| Tertiary/Exploratory |  |  |  |
| To identify and describe the barriers and facilitators of iCARE intervention readiness and implementation across study sites and to describe intervention costs | This objective is exploratory and descriptive in nature, therefore there are no specific endpoints | N/A | N/A |

**Study 2: HIV Treatment Intervention**

| OBJECTIVES | ENDPOINTS | JUSTIFICATION FOR ENDPOINTS | PUTATIVE MECHANISMS OF ACTION |
| --- | --- | --- | --- |
| Primary |  |  |  |
| To determine the efficacy of the combination of SMS + Peer Navigation to improve viral suppression among youth receiving ART across multiple sites | Viral suppression, defined as viral load <200 copies/mL at week 48 of intervention exposure | Viral suppression to levels below 200 copies/mL is a commonly used measure of successful ART. In addition, the public health tenet of U=U is based on evidence that HIV transmission does not occur when viral load is < 200 copies/mL. In addition, viral suppression is one of the indicators included in the UNAIDS 95-95-95 declaration | The investigational intervention will improve adherence to ART which will in turn increase the odds of achieving and/or maintaining viral suppression |
| Secondary |  |  |  |
| To determine the effect of the intervention on medication adherence  To determine the effect of the intervention on retention in care. | ART adherence as measured by:  a) Pharmacy drug pick-up (90% of days with medication) at week 48  b) Antiretroviral (ARV) drug concentration in Dried Blood Spot sufficient for viral suppression at week 48  c) Self report on a visual analogue scale of 90% at week 48  Retention defined as at least two care (non-study) visits, including youth clinic, in the prior 24-week period at week 48 | Pharmacy drug pick -up records and ARV drug concentration are objective indirect measures of adherence while self-report is a complimentary subjective measure.  Retention in care is a surrogate marker of treatment success | Practical assistance and other support provided by peer navigation combined with text message reminders lead to ART adherence  Practical assistance and other support provided by peer navigation will lead to retention in care |
| Tertiary/Exploratory |  |  |  |
| A tertiary objective is to test ART adherence and retention in care as mediators of the intervention effect AND age, gender, and mode of transmission (perinatal vs. behavioral) as potential moderators of the effect across multiple sites  An exploratory objective is to identify and describe the barriers and facilitators of iCARE intervention readiness and implementation across multiple sites and to describe intervention costs | Statistically significant (P<.05) mediation and/or moderation of the intervention effect on viral suppression  This objective is exploratory and descriptive in nature, therefore there are no specific endpoints | Evidence of mediation of the intervention effect provides an indication of how the effect is transferred to changes in viral load suppression. Differences in the intervention effect by subgroups of age, gender and mode of transmission indicate for whom the intervention works.  N/A | The investigational intervention will improve adherence to ART and retention in care, which will in turn lead to viral suppression, the effect of which may differ by age, gender and mode of transmission  N/A |

# STUDY DESIGN

## Overall Design

Two randomized stepped wedge trials will be conducted to investigate two interventions (each with mHealth + Peer Navigation components). A stepped wedge design is a type of cluster-randomized design in which clusters are randomized to one of several different sequences which dictate when the cluster will switch from a control condition to an intervention condition. An advantage of this design is that all clusters receive the intervention and individual-level randomization is not required. This is a pragmatic design that is typically used to evaluate how interventions would work in real-world settings. Study 1 will test a combination intervention that targets HIV testing and linkage to care while Study 2 will test a combination intervention that targets HIV treatment outcomes (retention, adherence and viral suppression). These studies will be implemented concurrently. The exploratory, implementation science objective, to identify and describe the implementation barriers and facilitators of both iCARE interventions across all study sites will be studied using mixed methods, which include data collection via closed-ended questionnaires, as well as key informant and focus group interview data collection.

**In Study 1 (HIV Testing and Linkage to Care)**, we hypothesize that using the same primary outcome as in the UG3 phase, the combination intervention, will remain efficacious across multiple sites. With the combination intervention of Social Media + Peer Navigation, i) the change in HIV seroprevalence in the intervention versus control period will be OR≥2.0. We will continue the intervention in each site after the intervention period to explore longer-term uptake and efficacy of the intervention.

| **STUDY 1 – HIV TESTING** | **~Year 01^a^** | | **~Year 02** | | **~Year 03** | |
| --- | --- | --- | --- | --- | --- | --- |
| **STUDY WEEKS** | **-24-0** | **0-24** | **25-48** | **49-72** | **73-96** | **97-120** |
| **TIME PERIOD** | **TIME 0** | **TIME 1** | **TIME 2** | **TIME 3** | **TIME 4** | **TIME 5** |
| Cluster 1: Ibadan (not randomized) |  |  |  |  |  |  |
| Cluster 3: RANDOM SELECTION (Jos+Sagamu vs. Lagos) |  |  |  |  |  |  |
| Cluster 2: RANDOM SELECTION (Jos+Sagamu vs. Lagos) |  |  |  |  |  |  |
| ^a^ Study years are approximate given the use of 24 week intervals | | | | | | |

| Study Start-Up Phase |  |
| --- | --- |
| Pre-intervention Phase |  |
| Intervention Phase |  |
| Post-Intervention Phase |  |

Trained Testing Peer Navigators (including an Outreach Coordinator) will conduct HIV testing outreach to at-risk youth using social media platforms to promote HIV testing. The Outreach Coordinator and Peer Navigators will be male or female young adults who are staff members (not volunteers), with experience working with key populations. For youths who express an interest in HIV testing, the peer will navigate them based on the youth’s preference to any of the local HIV testing locations or the peer navigator will perform the HIV test wherever the participant feels most comfortable (e.g., home, community location, clinic or office). Youth who are navigated to HIV testing will be asked to complete an optional, brief, anonymous and self-reported questionnaire regarding their demographic characteristics, HIV testing and risk behavior, and satisfaction with the intervention. Those diagnosed with HIV will be linked to their preferred HIV treatment clinic. While peer navigators will include youth men and women, the primary target of this study is YMSM.

To determine efficacy of the combination intervention, the change in HIV seroprevalence will be compared between the intervention and control period. HIV testing data for all young men as opposed to YMSM alone will be utilized mainly because HIV testing records do not routinely contain valid data on same-sex behavior.

As a secondary objective, we will assess the percentage of newly diagnosed youth who are linked to care for ART. As an exploratory objective we will assess barriers and facilitators of iCARE intervention readiness and implementation across multiple sites and describe intervention costs.

In addition, we will describe the seroprevalence of tested young men after the intervention period, which will provide an assessment of longer-term uptake of the intervention.

**In Study 2 (HIV Treatment Intervention),** the combination of Short Message Service (SMS; via adaptation of the TXTXT intervention) + Peer Navigation will be investigated to evaluate their effect on HIV treatment outcomes (viral suppression, ART adherence, and retention in care) across all sites. We hypothesize that viral suppression (defined as < 200 copies/mL) will be greater during the intervention period relative to the control period, reflecting an OR≥1.5. After the end of the intervention period, in each site, we will continue to measure outcomes via medical records abstraction to explore the longer-term durability of the intervention effect.

| **STUDY 2 – HIV TREATMENT** | **~Year 01^a^** | | **~Year 02** | | **~Year 03** | |
| --- | --- | --- | --- | --- | --- | --- |
| **STUDY WEEKS** | **-24-0** | **0-24** | **25-48** | **49-72** | **73-96** | **97-120** |
| **TIME PERIOD** | **TIME 0** | **TIME 1** | **TIME 2** | **TIME 3** | **TIME 4** | **TIME 5** |
| Cluster 1: Ibadan (not randomized) |  |  |  |  |  |  |
| Cluster 2: RANDOM SELECTION (Jos+Sagamu vs. Lagos) |  |  |  |  |  |  |
| Cluster 3: RANDOM SELECTION (Jos+Sagamu vs. Lagos) |  |  |  |  |  |  |
| ^a^ Study years are approximate, given the use of 24 week intervals | | | | | | |

| Study Start-Up Phase |  |
| --- | --- |
| Pre-Intervention Phase |  |
| Intervention Phase |  |
| Post-Intervention Phase |  |

We will enroll approximately 560 youth in total, ages 15-24 at the six sites (including virologically suppressed and viremic individuals) with HIV regardless of gender, who have been on ART for at least 3 months at the HIV clinics in IDI, LUTH, NIMR, LASUTH, JUTH, Sagamu or their satellite clinics. Enrollment will be monitored to ensure a representative proportion of suppressed versus viremic participants at study entry.

Trained Treatment Peer Navigators (one for 5 participants) will be selected from the clinics using the following criteria: i) on ART, ii) virologically suppressed with viral load <200 copies/mL based on viral load in the preceding 12 months, if available, iii) 18 to 30 years of age, and iv) “doing well” according to their physician’s subjective assessment. Peer Navigators will be carefully selected to represent diversity in each clinic population including by religion, primary language, gender, age and other important characteristics. Each Peer Navigator will navigate 5 youths, by providing practical assistance and other support to optimize adherence. Provided support will be determined through a structured Needs Assessment process that will be conducted immediately prior to beginning the intervention. In addition, peer navigators will foster a sense of community among the participants in their respective groups. Participants will receive SMS text messages, adapted from the CDC recommended TXTXT, which includes daily, free, bi-directional and personalized text messages, delivered using a secure platform provided by Dimagi CommCare. The SMS will be personalized to remind and encourage the youth to adhere to their ART. Each participant will be exposed to the intervention for 48 weeks.

The primary efficacy measure for this combination intervention is the proportion of participants with viral load <200 copies/mL during the intervention period in comparison to the control period. Those with viral load ≥200 copies/ml at study entry, or at weeks 24 and/or 48 of intervention exposure, may have resistance testing done to better understand their viral non-suppression. In addition, per the clinical standard of care, youth with persistent viremia despite enhanced adherence counselling will be reviewed for change to second or third line ART regimens.

The secondary measures of efficacy are: i) adherence (based on pharmacy pick up records, self-report, and DBS drug levels) and, ii) retention in care based on medical records during the intervention period in comparison to the control period.

We will explore the implementation science objective, to identify and describe the implementation barriers and facilitators of both iCARE interventions across all study sites using mixed methods, which include data collection via closed-ended questionnaires, as well as key informant and focus group interview data collection. In addition, we will continue to abstract primary and secondary outcomes, from the medical record after the intervention period to describe the durability of the intervention effect.

## Scientific Rationale for Study Design

Evidence supports the combination of peer navigation and SMS reminders to improve HIV outcomes. In South African adults, Steward and colleagues used a cluster 3-arm randomized controlled trial (SOC/Standard of Care vs. SMS only vs. SMS + peer navigation) to evaluate the value of a combined intervention using peer navigation and SMS check-in messages in improving HIV treatment outcomes.^32^ In the study, peers were trained to help other patients navigate HIV care and prevention through a minimum of monthly in-person meetings, phone and SMS contact as needed (minimum biweekly check-ins) and accompanying patients to clinic appointments as needed.^33,34^ In addition to the SMS text check-in reminder with peer navigators, patients in the combination intervention arm also received SMS text “healthy living” reminders as well as reminders for clinic appointments. In comparison to the SOC arm, participants in SMS + peer navigation arm were almost twice as likely (OR 1.83, p<0.01) to be retained in care at 12 months (with retention defined for those on ART as at least 4 care visits within 12 months and for those not on ART defined as 2 or more visits in 12 months).^32^ There was no significant difference in retention between the SMS alone vs. SOC arms. Consistent with this finding in adults, SMS alone failed to improve adherence to ART and co- trimoxazole prophylaxis in the only randomized trial of this intervention in youth\recently reported from Uganda.^35^ These data suggest that combination interventions may be necessary in youth. Indeed, the combination of SMS text + peer navigation may be particularly suited to youth because it aligns with their developmental and social needs for peer engagement & acceptance and affinity for telecommunication, thus holding promise across the HIV care continuum.

Results from the UG3 phase of the iCARE study suggest both preliminary efficacy and feasibly of our combination intervention approach. In the HIV testing arm, during the 12-month intervention period, the iCARE intervention increased by >30% the number of HIV tests completed among the young men, ages 15-24 at IDI/CoMUI and increased the seropositivity rate among cases to 4.9%, compared to 1.7% in the 6-month period prior to the intervention. In the HIV treatment arm, the proportion of patients with a suppressed viral load (VL<200 copies/mL) increased by 71% over the 48-week intervention period, from 35% at baseline to 60% at 48 weeks, a statistically significant change (McNemar test, OR = 6.00, p = 0.013). Self-reported adherence ($\geq$90%) increased from baseline at both 24 weeks (63%, p = 0.008) and 48 weeks (68%, p = 0.031). Medication possession ration (MPR) $\geq$90% increased numerically at both weeks 24 and 48 (85% and 80%, respectively), achieving statistical significance at 24 weeks only (p = 0.022). Retention in care at 48 weeks was 87.5%. Satisfaction as rated by individuals who completed HIV tests and participants in the HIV treatment intervention was over 90%.

## Justification for Intervention

Youth, particularly YMSM, are weak links in Nigeria’s response to the HIV epidemic. While there are many complicated factors that fuel the epidemic in these groups, a void of evidence-based interventions is remediable and necessary to advance the UNAIDS 95-95-95 goals. Therefore, we will investigate evidence-based youth-specific approaches that include peer navigation and mHealth components, which have shown initial evidence of efficacy and feasibility in the UG3 pilot, across multiple sites.

## End-of-Study Definition

These studies are part of the second (UH3) phase of the iCARE, Nigeria study. If the hypotheses of this UH3 phase are met, the sustainability plan will be activated to maintain the intervention beyond the study context.

# STUDY POPULATION

The population for Study 1 is the total number of HIV tests completed, and resulting HIV seroprevalence, among youth in/around Ibadan, Lagos, and Jos.

The study population for Study 2 comprise youth living with HIV and receiving antiretroviral therapy (ART) at IDI, LUTH, NIMR, LASUTH, OOUTH Sagamu, and JUTH and their satellite clinics.

## Inclusion Criteria

**Study 1 (Testing and Linkage to Care)**

HIV tests of young men ages 15-24 residing in Ibadan, Lagos, Jos and surrounding areas in the pre-intervention period (24 to 72 weeks prior to the launch of the intervention in each site, depending on randomization) and during the intervention period (48 weeks) will be abstracted from surveillance records of each clinic for analysis. In addition, HIV testing records for sexual partners of eligible cases, tested via iCARE (i.e., per national partner testing guidelines), will also be abstracted regardless of sex or age.

**NB:** For the HIV testing and linkage intervention, no human subjects will be enrolled and no identifying data will be collected. Routine HIV testing data from surveillance records at each site/clinic will be abstracted in a de-identified format. Demographic, behavioral and intervention satisfaction data will be collected in anonymous format for eligible cases and their sexual partners.

**Study 2 (Treatment Intervention)**

Inclusion criteria:

1. Age 15-24 years; if 15 years old must have parental consent or be emancipated i.e., has been granted status of adulthood by a court order, has lived independent of parental guidance for at least a year, is married, living on the street, or is head of a household
2. Living with HIV infection
3. Registered in the study clinics or their satellite clinics
4. On ART for at least 3 months. Participants may be virologically suppressed or viremic/failing on ART. We will monitor enrollment to ensure a representative proportion of suppressed versus viremic participants*.*
5. Understands and can read basic English, Hausa, Pidgin English, or Yoruba
6. Willing and able to provide informed assent or consent.
7. Intention to remain a study clinic patient during the study observation and intervention period (up to 96 weeks).

**NB:** There are no eligibility criteria specific to ART adherence or retention since these are subject to variation over time in youths, cell phone ownership is not required; cell phones will be provided to participants without one.

## Exclusion Criteria

Study 2: Exclusion criteria

1. Inability to provide informed assent or consent.
2. Youths who are 15 years old and do not receive parental consent or are not emancipated

## Lifestyle Considerations

N/A

## Screen Failures

Participants who do not meet the eligibility criteria will not be enrolled into the study.

## Strategies for Recruitment and Retention

The HIV testing and linkage study will reach at-risk youth through social media and in-person outreach conducted by peer navigators, who will be trained to implement the manualized combination intervention, focused on promoting HIV testing and linkage to care.

In the HIV treatment outcomes study, a sampling frame will be created for eligible patients from medical records at each of the six sites (and satellite sites) and stratified by status as virally suppressed versus viremic. Eligible patients will be contacted from each stratum or approached in clinic and screened for eligibility until the accrual target is reached, while ensuring a representative proportion of suppressed versus viremic participants.

A screening log will be maintained to track ineligible cases and reasons for ineligibility, as well as refusals and no-shows.

# STUDY INTERVENTION(S) OR EXPERIMENTAL MANIPULATION(S)

## Study Intervention(s) or Experimental Manipulation(s) Administration

**Study 1: (HIV testing and linkage to care Intervention)** includes social media engagement and peer navigation.

Social media engagement: Outreach and testing will be done using a targeted approach. Trained HIV Testing Peer Navigators (male and female youth) will conduct outreach and engagement on social media platforms that are used and trusted by YMSM (e.g., Whatsapp, Grindr, Facebook). Testing Peer Navigator’s activities will include using existing or creating new closed groups on these platforms, plus respondent-driven new invitations in order to generate a following. The Outreach Coordinator and Testing Peer Navigators will be male or female young adults who are regular staff (not volunteers), with experience working with key populations.

Prior to deployment, the Testing Peer Navigators will be trained on 1) profile set-up; 2) scripts for messaging, and instructions for structuring the interaction (e.g., how many times to message before moving on, providing responses as quickly as possible); 3) FAQs to guide unscripted interaction (e.g., Don’t exchange personal information/photos; Do stick to pre-approved themes). The social media interactions will promote HIV testing.

Peer navigation: Testing Peer Navigators will also be trained to conduct community-based outreach and HIV testing, with emphasis placed on flexibility, building trust and maintaining confidentiality. In addition, they will be trained in pre and post-test counseling and in the management of reactions to the test results. HIV testing will be promoted via social media and in-person interactions, e.g., at locations where young people may socialize locally. Social media messaging by Testing Peer Navigators will focus on the benefits of HIV testing, management of stigmatization of HIV testing, and peer-based social support. The navigator will emphasize the advantages of HIV tests, e.g., “know your status,” autonomy for self-management, benefits of early diagnosis and treatment. In addition, the navigator will emphasize free testing and confidentiality of treatment.

For individuals who express interest in HIV testing, the Testing Peer Navigators will navigate them based on the youth’s preference to either i) IDI/CoMUI, LUTH, NIMR, LASUTH, OOUTH Sagamu, or JUTH testing site, ii) another testing site that the youth prefers, or iii) HIV test performed by the peer navigator at a location the youth finds comfortable. Youth who are navigated to HIV testing will be asked to complete an optional, brief, anonymous and self-reported questionnaire regarding their demographic characteristics, HIV testing and risk behavior, and satisfaction with the intervention. Youth who have a negative test result will be counseled to return for follow-up testing and reminded to do so according to the national guidelines and local surveillance practices. Youth with a positive HIV test will be navigated to the IDI/CoMUI, LUTH, NIMR, LASUTH, OOUTH Sagamu, or JUTH clinic (or any other treatment site preferred by the youth) for confirmatory testing and linked to care for ART initiation according to national guidelines.

**Study 2: HIV treatment intervention (retention, ART adherence and viral suppression) Intervention,** also includes two components- peer navigation and SMS text messaging

Peer Navigation: The Treatment Intervention Peer Navigators in this pilot study are defined as HIV positive, medication adherent role models living with a shared experience and a shared community membership as the populations with whom they will work. The Treatment Peer Navigators will be selected from the IDI, LUTH, NIMR, LASUTH, OOUTH Sagamu and JUTH, or their satellite clinics using the following criteria: i) HIV-1 viral load < 200 copies/mL within the preceding 12 months (if available), ii) subjectively assessed as “doing well” by their physician, based on the categories- doing well, doing poorly, or variable, iii) willingness to volunteer as navigator, iv) successful completion of comprehensive training that includes modules on privacy and confidentiality. A designated clinic/study staff, assisted by the Treatment Peer Navigator, will conduct a Needs Assessment and ART education for participants. The navigator will initiate a minimum of two encounters per month (at least once every two weeks) with each of their assigned participants (in person or by texting/voice call/social media).

Treatment Peer Navigators are tasked with optimizing treatment outcomes by:

1) Assessing and addressing barriers to care engagement (e.g., transportation to clinic).

2) Providing referrals and linkage to supportive services.

3) Attending appointments with participants as needed.

4) Enabling early detection of those at risk of poor outcomes; and

5) Fostering community and providing supportive care (e.g., medication adherence support, support for disclosure of HIV status).

Each Treatment Peer Navigator will meet in person or by phone at least every two weeks with a designated clinic staff to discuss emerging issues with their assigned peers and formulate practical solutions including allocation of funds to participants based on the findings of the Needs Assessment. This meeting may occur in a group-based format with other navigators.

Funds that are available to the clinic, and are not study-provided (e.g., through philanthropy or indigent fund), may be used to address some of the needs of study participants, based on availability. Peer navigators will receive a small stipend, which includes funds for their transportation/communication costs.

SMS text message reminders: This study will utilize a locally adapted version of our CDC-recommended, TXTXT intervention.^17^ Messaging will consist of daily, bi-directional and personalized text messages that will be delivered, using a platform provided by Dimagi CommCare. All text-related data will be securely stored by Dimagi per their privacy policy. The SMS messages will promote adherence and the content will be selected by and for the participant themselves with consideration given to each person’s need for privacy and confidentiality and timed to coincide with individual dosing schedule.

Trained Treatment Peer Navigators will use a structured text message tailoring form to elicit message content and then set up automated messages in the Dimagi platform, i.e., set-up daily reminders according to the participants’ dosing schedule and preferred message content. To protect privacy and confidentiality, we will encourage participants to delete text messages after taking medication, to use confidential messages that do not reveal HIV status or mention medications, and we will encourage them to use a passcode on their phone to protect confidentiality. Participants will have the option to choose a personalized message that may be changed as requested throughout the study period.

Bi-directionality is reflected in a series of follow-up messages. Participants will be asked to send a text message response indicating they have successfully taken their ART per schedule. An automated response system provides options for responding, including: 1) “Yes” or 2) “No.” If the participant responds, “Yes” an affirmative and encouraging message will be sent in reply (e.g., “Well done!”); a “No” response will trigger an acknowledging and encouraging message (e.g., “You can do it!”). This 2-way message system provides a positive feedback to promote motivation, self-efficacy, and support.

Participants will use their own cell phones for receipt of messages, however, we will provide phones to those who do not have them (estimated to be approximately 40% of clinic population based on UG3 findings), to ensure broad participation. Peer navigator will maintain logs of contacts with participants and phone-related problems

## Fidelity

Intervention fidelity will be determined by the proportion of intervention tasks completed, using a fidelity checklist.

### Interventionist Training and Tracking

All Peer Navigators will undergo training prior to deployment. The training curriculum for research staff includes human subjects education, privacy and confidentiality, participant safety, and intervention and data collection training.

## Measures to Minimize Bias: Randomization and Blinding

In the stepped wedge design, the Ibadan site will not be randomized because it already began the intervention in the UG3 period, however, the other study sites will be randomized in two clusters: one cluster will include the Lagos sites (LASUTH, LUTH, NIMR) and the other cluster will include OOUTH Sagamu and JUTH. Randomization of the two remaining clusters will reduce bias for observation of the control period in comparison to the intervention period. The randomization assignments will be generated by computer algorithm by the study statistician and will remain concealed until after data has been collected in the observation period, before launch of the intervention in randomized sites.

## Study Intervention/Experimental Manipulation Adherence

N/A

## Concomitant Therapy

N/A

# STUDY INTERVENTION/EXPERIMENTAL MANIPULATION DISCONTINUATION AND PARTICIPANT DISCONTINUATION/WITHDRAWAL

## Discontinuation of Study Intervention/Experimental Manipulation

The study will terminate upon completion of the funding period unless a no-cost extension is secured or premature termination is mandated by the IRB, the NICHD, or other constituted authority.

## Participant Discontinuation/Withdrawal from the Study

Participants may withdraw or be discontinued from the Study 2 at any time for any of the following reasons:

1. Withdrawal of consent for any reason
2. Potential for harm to the participant in the opinion of the site investigator or their physician

Peer navigators in either of the studies will be discharged if there are concerns about their conduct on study, including violation of privacy and confidentiality requirements. Peer navigators who quit the study prematurely will be replaced.

## Lost to Follow-Up

Participants will be considered lost to follow-up in Study 2 if they do not complete the week 48 visit of the intervention period. Measures to be taken by Treatment Peer Navigators to prevent loss to follow-up are similar to those already in place in the site clinics, including maintaining contact information up-to-date, using multiple methods of contact, and providing reminders for study visits.

# STUDY ASSESSMENTS AND PROCEDURES

## Endpoint and Other Non-Safety Assessments

**Study 1 (HIV Testing and Linkage to Care)**

Study HIV Testing data will be collected in 24-week increments for up to 96 weeks during the study period.

The primary endpoints for the combination intervention for HIV testing/linkage study is the HIV seroprevalence, calculated as the number of confirmed HIV cases divided by the total number of tests during the intervention versus control period. To achieve this, HIV testing data for all young men, ages 15-24, will be abstracted from surveillance records at each site in each observation and intervention periods.

Secondary endpoints include linkage to care of newly HIV diagnosed youth who undergo testing through IDI, LUTH, NIMR, LASUTH, OUTH Sagamu, and JUTH (i.e. tested on site at the clinic or by a Peer Navigator during community outreach). This is defined as registering and completing a clinic visit where ART or opportunistic infection treatment is provided within 30 days of a confirmatory HIV test. These data will be captured in HIV testing encounter forms used for surveillance purposes and tracked using an anonymous identification number.

The anonymous self-reported demographic, behavior, and satisfaction questionnaire will be collected in paper-and-pencil format or (as connectivity allows), deployed on a mobile device via the secure data capture system, REDCap, hosted at Northwestern University. Aggregate social media tracking data and HIV testing encounter data will be entered into REDCap and stored for analysis.

**Study 2 (HIV Treatment Intervention)**

Study data will be collected at enrollment and at 24-week increments during the study period, up to 96 weeks per the stepped wedge design (See Schedule of Events, Section 1.3)

The primary endpoint for the HIV treatment intervention study is viral suppression defined as HIV < 200 copies/mL during the intervention period versus control period. We will collect blood via venipuncture for HIV viral load quantification at all study visits. Two tubes of blood, each 8-10 mL (total 16-20 mL) will be collected. One tube will be used for viral load, dried blood spot (DBS), and CD4 quantification (CD4 completed at the first study visit only); the other tube will be stored for viral resistance testing (select samples) and future potential studies. Viral load and CD4 quantification will be performed at designated HIV laboratories. Additional abstraction of viral load lab values from the medical records will occur post-intervention, through 120 weeks, to assess the duration of the intervention effect.

Secondary endpoints will be captured to measure both secondary effects and mediation of the intervention effect and include:

1. Adherence based on:

- Dried Blood Spot (DBS): From one tube of the collected blood sample (i.e., 8-10 mL), five 50µL spots on protein saver cards will be used for measurement of ART levels. The samples will be dried out at room temperature for 24 hours, then sealed tightly with desiccant and a humidity indicator, and stored at -20 degrees Celsius until analysis.
- ART pharmacy pick-up: Pharmacy records of ART pick-up are captured as part of routine care at each clinical site and will be abstracted from the medical record at all study visits (for the 24-week period prior to the visit). Additional data abstraction will occur post-intervention, through 120 weeks, to assess the duration of the intervention effect. These data will be used for calculation of MPR.
- Self-report of ART adherence via 30-day visual analogue scale (VAS), 30-day number of missed doses, and 4-day missed doses.^36^

1. Retention in care

- This is defined as at least two care (non-study) visits, including youth club visits, to the clinic in a 24 week period. Visit dates are captured as part of routine care at each clinical site and will be abstracted from the medical record at all study visits (for the 24 week period prior to the visit). Eligible visits include “Youth Club” visits that take place in the HIV clinic or any other non-study-mandated visit. Additional data abstraction will occur post-intervention, through 120 weeks, to assess the duration of the intervention.^32^

Other assessments

1. Implementation Assessment

Anticipated variations in local practices, strengths and challenges at intervention sites will create an opportunity to measure and understand: 1) how the interventions are implemented across the study sites and reflect key study outcomes, and 2) the contextual factors that impact the efficacy of the intervention. We will use the RE-AIM framework to evaluate our implementation strategies,^37^ and explore implementation contextual factors using relevant domains from the Consolidated Framework for Implementation Research (CFIR).^38^ These frameworks were chosen based on relevance and have been used in low and middle income countries, and in evaluating and understanding the success and challenges of implementation to accelerate success to achieve 95-95-95^39^

We will assess indicators of intervention implementation, including satisfaction and acceptability^40^ among study participants via study questionnaire data. Additional indicators of implementation feasibility, reach, and adoption, as well as study cost will be collected from administrative records and tracking logs. Finally, questionnaire, key informant, and focus group data will be collected with study staff and clinic staff, as well as representatives from community-based organization (CBOs) before, during, and after the implementation of the intervention in each site to elicit perceptions of barriers and facilitators of intervention implementation. These findings will inform practice-based implementation and sustainability after the end of the study.

1. Demographic, Medical History & Psychosocial Assessments

Demographic and medical history data, including age, gender and mode of infection will be collected via study participant computer assisted personal interviewing (CAPI) questionnaires and via medical records abstraction to describe the sample and test moderators of the intervention effect. In addition, psychological assessments will be collected to fully describe characteristics often associated with HIV outcomes, including measures of HIV treatment knowledge,^41^ HIV medication self-efficacy^42^, HIV stigma,^43^ depressive symptoms,^44^ alcohol and drug use^45^.

## Safety Assessments

Participant safety will be monitored through weekly review of peer navigation activity logs and encounter forms and monthly tallies of completed satisfaction questionnaires. In addition, study staff will be trained to report concerns about safety directly to the local Principal Investigator at each site. If any study staff member discovers any untreated condition (e.g., onset of physical or mental health condition), they will refer the participant for appropriate treatment immediately. The study multiple Principal Investigators at the lead site (Profs. Robert Garofalo and Babafemi Taiwo) will be informed of any significant safety concerns within 24 hours of the site PI becoming aware of the event. Participant safety will be discussed at weekly all team research meetings, and a written log of all safety events will be maintained.

Since breach of confidentiality is a potential safety concern in these studies, all study personnel and peer navigators will be trained regarding privacy and confidentiality. The training will include reviewing possible scenarios and key questions to assess risk. We will train staff to err on the side of caution and to contact the clinical supervisor or site investigator as needed. Under the guidance and direction of clinical supervisors (or site investigator), study staff will be trained when, if appropriate, to escort participants to the nearest emergency room or clinic in the event of an emergency.

## Adverse Events and Serious Adverse Events

### Definition of ADVERSE EVENTS AND Serious Adverse Events

While an adverse event (AE) is typically defined as any untoward medical occurrence in a participant enrolled in a study, for the purpose of these studies in which no study drugs are provided or clinical care rendered, reportable AEs are limited to the events of special interest in section 8.2.7.

A serious adverse event (SAE) is one that meets one or more of the following criteria:

- Results in death
- Is life-threatening (places the subject at immediate risk of death from the event as it occurred)
- Results in inpatient hospitalization or prolongation of existing hospitalization
- Results in a persistent or significant disability or incapacity
- Results in a congenital anomaly or birth defect
- An important medical event that may not result in death, be life threatening, or require hospitalization may be considered an SAE when, based upon appropriate medical judgment, the event may jeopardize the participant and may require medical or surgical intervention to prevent one of the outcomes listed in this definition.

### Classification of an Adverse Event

AEs and SAEs will be classified by the site investigator as related or not related to the study.

### Time Period and Frequency for Event Assessment and Follow-Up

Unanticipated problems will be tracked in problem and adverse event logs throughout the study and managed according to local IRB or ethics board requirements.

### Adverse Event Reporting

AEs, including SAEs, will be collated and presented as part of the study reports.

### Serious Adverse Event Reporting

All SAEs will be reported to the Northwestern team and the NIH Program Officer within 3 days of occurrence. SAEs that are unanticipated and related or possibly related to the study will be reported to the IRB and the DSMB within 7 working days of the study team becoming aware of its occurrence.

### Reporting Events to Participants

In consultation with the site IRB(s) and the NIH Program Officer, the study team will notify participants and/or implement modifications to the study protocol.

### Events of Special Interest

Potential AEs include inadvertent disclosure of HIV status and significant negative emotional or social consequences of interactions and interventions that occur during the study. In addition, though unlikely based on findings of the recent UG3 phase, there is a risk of arrest, incarceration or similar legal jeopardy as a result of the social and legal environment for young men who have sex with men. These events will be reported as outlined in 8.2.1, 8.2.4 and 8.2.5

Disruptions

Disruptions to a research study can be brought on by a multitude of unpredictable events, i.e. political unrest, strikes, disease outbreaks. When such an event occurs, the local PI at each site will meet with the Co-Investigators and other study staff to formulate a plan to continue study data collection, while ensuring the safety of study participants. The study MPIs (Drs. Taiwo, Garofalo) will be informed of any significant disruptions within 24 hours of the site PI becoming aware of the event. In some cases, the necessary changes may require deviating from the study protocol. Participants should be kept informed of changes to the study that could impact them. The sites will report all such events to their IRB and sponsoring agencies.

### Reporting of Pregnancy

Pregnancies that occur in the course of the study will be described in study reports. Since ART is not provided in this study, pregnancy outcomes will not be tracked by the study team.

## Unanticipated Problems

### Definition of Unanticipated Problems

Unanticipated problems involving risks to subjects or others include, in general, any incident, experience, or outcome that meets **all** of the following criteria:

- Unexpected in terms of nature, severity, or frequency given (a) the research procedures that are described in the protocol-related documents, such as the IRB-approved research protocol and informed consent documents; and (b) the characteristics of the subject population being studied;
- Related or possibly related to participation in the research (“possibly related” means there is a reasonable possibility that the incident, experience, or outcome may have been caused by the procedures involved in the research); and
- Suggests that the research places subjects or others at a greater risk of harm (including physical, psychological, economic, or social harm) than was previously known or recognized.

### Unanticipated Problems Reporting

All events that meet the criteria for an unanticipated problem will be reported to the site IRB and the NIH Program Officer within 7 days of the study team becoming aware of its occurrence.

### Reporting Unanticipated Problems to Participants

In consultation with the IRB and Program Officer, the study team will implement participant notification and/or modifications to the study protocol.

# STATISTICAL CONSIDERATIONS

## Statistical Hypotheses

**Study 1: HIV Testing and Linkage to Care**

Primary Endpoints:

HIV Testing Hypotheses: the effect size in HIV seroprevalence during the intervention versus control period will be OR≥2.0.

Secondary Endpoints:

Linkage to Care Hypothesis: There will be at least 90% linkage to care among those with newly diagnosed HIV

**Study 2 HIV Treatment Intervention**

Primary Endpoint:

We hypothesize that the rate of viral suppression (defined as < 200 copies/mL) during intervention period will be greater relative to control period, reflecting an OR≥1.5.

Secondary Endpoints:

Retention in Care Hypothesis: Retention in care during the intervention period will be greater relative to the control period. Eligible visits include the monthly “Youth Club” that takes place in the HIV clinic or any other non-study-mandated visit.

Adherence Hypothesis: Adherence will be greater during the intervention period in comparison to the control period. Adherence will be compared both for ART concentration, via dried blood spot, pharmacy pick-up records (100 minus percentage of days alive but without medication), and self-report (30-day visual analogue scale adherence, 4-day missed doses)

Tertiary Endpoint:

Medication adherence and retention in care will mediate the intervention effect on viral load suppression. We further hypothesize that there will be no significant moderation effect by age, gender and mode of infection, that is, the intervention effect in subgroups will be not be of differing strength or directionality.

## Sample Size Determination

Power analysis For Study 1, small to moderate correlation within clusters (ICC = 0.10), there is sufficient power (0.805) to detect the OR of 2.0 in positivity rate with 315 tests completed on average in each cluster. For Study 2, with a small to moderate within cluster correlation and moderate individual auto-correlation (0.50), this analysis will have adequate power (power = 0.945) to detect an OR of 1.5 in the proportion of participants achieving viral suppression with at least 90 participants per site.

## Populations for Analyses

The population for Study 1 is the total number of HIV tests completed, and resulting seroprevalence, among youth in/around Ibadan, Lagos, and Jos.

For Study 2, the population will include youth on ART at the IDI, LUTH, NIMR, LASUTH, JUTH, OOUTH Sagamu and their satellite clinics.

## Statistical Analyses

### General Approach

For descriptive statistics, percentages will be presented for dichotomous outcomes (e.g., viral suppression vs. non-suppression) and means will be presented for continuous outcomes (e.g., medication adherence). Similarly, changes between intervention and control periods will be summarized via odds ratios. For Study 1, generalized linear models (GLM) will be used. For Study 2, generalized estimating equations (GEE) will be used to account for multiple observations of participants. For both studies, a binomial distribution and logit link function will be used to evaluate the statistical significance of the intervention effect, while controlling for secular trends in the outcomes over time and clustering within clusters. This analysis will follow the principles of intention to treat analysis and will be evaluated by examining the statistical significance of the regression coefficient for the intervention on the two primary outcomes of the studies. For differences in HIV seroprevalence in Study 1 and differences in viral load suppression in Study 2, we will compare intervention and control periods at the end of the intervention period, i.e. 48 weeks.

### Analysis of the Primary Endpoint(s)

**Study 1**

A GLM will be used to estimate difference in seroprevalence between intervention and control periods by examining the statistical significance of the intervention effect (i.e., identifying interventions periods), with a fixed effect for cluster, and a fixed categorical for fixed effect to account for secular changes in seropositive rates over time. An odds ratio of ≥2.0 will indicate satisfactory evidence of the intervention on seroprevalence.

**Study 2**

A GEE will be used to estimate the difference in viral suppression (<200 copies/mL) between intervention and control periods with participant as the cluster level effect, fixed effect for cluster, and a fixed categorical effect to account for secular changes in viral suppression over time. An odds ratio value ≥1.5 will be considered satisfactory evidence of efficacy of the intervention on viral suppression.

### Analysis of the Secondary Endpoint(s)

**Study 1**

Linkage to Care: To estimate the effect of the intervention on linkage to care we will compute the percentage of HIV positive individuals that are linked to care. GLMs will be used to compare intervention versus control periods and will be evaluated in identical models to those of the primary endpoints.

**Study 2:**

Retention in Care: To estimate the effect of intervention on retention in care we will examine we will analyze GEEs to account for multiple observations of participants and fixed effects to account for the clusters. We will compare change in the proportion of participants retained versus not retained, with retention defined as at least two clinic visits in the prior 24 weeks.

Adherence: To estimate the effect of the intervention on adherence we will examine GEEs to account for multiple observations of participants and fixed effects to account for the clusters. We will compare change in the proportion of participants adherent to ART based on 90% adherence on the VAS, number of days possessing medication and level of ART medication in DBS samples.

Mediation analyses

Mediation analysis will be conducted using a structural equation modeling to examine the indirect effect of the intervention on viral suppression via adherence. The statistical significance of the indirect effect will be evaluated to examine mediation. Cluster membership will be accounted for using a fixed effect.

### Safety Analyses

Safety issues that occur in the course of the studies will be described in annual reports to the IRBs and DSMB.

### Baseline Descriptive Statistics

We will describe age, gender, and mode of infection, as well as ethnicity/tribal affiliation, religion, employment, highest level of education, and marital/partnership status using measure of central tendency and dispersion.

### Planned Interim Analyses

Interim analysis of study endpoints will be performed at the end of year 2.

### Sub-Group Analyses

Moderation of the intervention effect will be analyzed by subgroups of age, gender and mode of infection. This will be evaluated by examining interaction effects between the fixed effect for the intervention and age, gender, and mode of infection as part of a GEE.

### Tabulation of Individual Participant Data

N/A

### Exploratory Analyses

Implementation assessment data from questionnaires and abstracted from administrative and programmatic records will be analyzed using descriptive statistics, including measures of central tendency and dispersion. As relevant, multivariate regression models accounting for clustering at the site level will be used to explore individual-level factors (of study participants and staff) associated with measures of implementation feasibility, acceptability and satisfaction. In addition, we will explore the relationship between implementation indicators and overall effectiveness and factors modifying these associations to understand variability between sites or subpopulations. Qualitative data from focus group and key informant interviews will be audio recorded, transcribed verbatim, and when needed, translated into English. Dedoose^46^ software will be used to store interview data and facilitate analysis using deductive and inductive content analysis. The coding structure will start with the CFIR domains relevant to the implementation and focus on understanding success, challenges and variability in key indicators of implementation.

# SUPPORTING DOCUMENTATION AND OPERATIONAL CONSIDERATIONS

## Regulatory, Ethical, and Study Oversight Considerations

### Informed Consent Process

In Study 1, while no individuals will be enrolled for the purposes of assessment of primary outcomes, individuals who complete HIV testing will be consented prior to completion of the brief demographic, behavioral and satisfaction survey (via a consent statement with waiver of documentation of consent).

For human subjects research in Study 2, no research data will be collected prior to informed assent or consent. Consent is conducted in a private room, by trained research staff. The consenting process will ensure autonomy of the youth by explicitly stating that participation in the research is voluntary, and that the youth is free to participate or withdraw at any time. The consenting procedure, among other elements, will cover: a) sources of potential harm (e.g., inadvertent disclosure even if the possibility is remote, or routine risks such as from blood draw); and b) importance of the knowledge to be gained (e.g. findings may improve understanding of how to improve the health for youths living with HIV).

While telephones will be provided to approximately 40% youths who do not have one (based on findings from the UG3) and a small sum will be made available to facilitate provision of practical support (based on needs assessment) in Study 2, there will no overt or covert coercion, enticement or intimidation of the participants.

All participants in the implementation assessment, including study staff and CBO representatives will be consented prior to participation using written consent (if identifying data are collected) or via a consent statement with waiver of documentation of consent (for anonymous data collection).

#### Consent/assent and Other Informational Documents Provided to participants

**Determining Capacity.** The cognitive capacity of youths will be carefully assessed as part of the consenting process. The trained Study Coordinator will proceed through each component of the consent, written to Nigerian primary school or below reading level, and will make an assessment of the youth’s decisional capacity to consent prior to signing. Potential participants will be asked questions designed to assess their capacity to understand what the study is about, appreciate, reason with, and express a choice about participation in the protocol.

**Parental consent**

Parental consent is required for unemancipated minors, but not for emancipated minors to participate in Study 2. In compliance with the 2014 Federal Ministry of Health Guidelines for Young Persons’ Participation in Research and Access to Sexual and Reproductive Health Services in Nigeria, youths 16 years and older or emancipated minors aged 15 years are exempt from parental consent. This guidance defines an emancipated minor as a person under the age of 18 years who has been granted status of adulthood by a court order, has lived independent of parental guidance of at least a year, is married, living on the street, or is head of a household.

Parental consent may decrease participation rates of emancipated minors because some youth will fear that their HIV status may be “outed” as a result of participation. Furthermore, the nature and scope of the proposed research do not pose more than “minimal risk” to participants. To compensate for waiver of parental consent or emancipated minors, minor participants receive a formal individual assessment of capacity to consent to ensure their understanding of study goals, procedures, and risks from disclosure of sensitive information.

#### Consent Procedures and Documentation

Informed consent as described above must be obtained and documented before any human subjects research. This applies to completion of the consent statement in Study 1 (HIV Testing Intervention) prior to the demographic, behavioral, and satisfaction questionnaire, and the assent or consent form for Study 2 (HIV Treatment Intervention), as well as consent for participation in the implementation assessment. Youths who are assented for participation with parental permission will be consented for participation at the next available visit after their sixteenth birthday.

### Study Discontinuation and Closure

The study will be discontinued when all data are complete, including HIV testing surveillance data in Study 1 and abstraction of HIV treatment data from medical records in Study 2, collected after the end of the intervention period in order to assess durability of the intervention effect.

### Confidentiality and Privacy

To protect the integrity of the participant’s data, staff will assign each individual a unique participant identification number (PID) at study enrollment. This code number will be used for all study data. We will maintain a list of participants with links between identifying information and code numbers to avoid any duplication in cases and to facilitate follow-up in Study 2. Only staff who have been trained in human subjects protection will have access to these lists, which will be kept on a secure server with password protected access. Consents are stored in a locked cabinet away from workstations and are only accessed in the event of a consent amendment or an audit. Locator files are kept in a locked cabinet in the research area, separate from data files, and are updated at each research visit.

### Future Use of Stored Specimens and Data

Drug resistance testing will be used to determine whether a participant with HIV has a mutated form of the virus that does not respond to ARTs, and as a result, could not have benefited from this intervention. We will collect and store venous blood and plasma at each study time point. Select participants will be identified and their samples sent to a reference laboratory for testing. Findings from resistance testing will be made available to the site and may be used to guide selection of an HIV regimen when initiating or changing ART and inform standard of care practices after the intervention period. We will store blood plasma samples indefinitely (with participant consent) for future studies as proposed by study investigators.

### Key Roles and Study Governance

| **Site** | **Site PI** | **Email Address** |
| --- | --- | --- |
| University of Ibadan/IDI | Prof. Olayinka Omigbodun | [olayinka.omigbodun@gmail.com](mailto:olayinka.omigbodun@gmail.com)  [fouryinkas@yahoo.co.uk](mailto:fouryinkas@yahoo.co.uk) |
| LUTH | Prof. Alani Sulaimon Akanmu | [asulaimona@gmail.com](mailto:asulaimona@gmail.com) |
| LASUTH | Dr. Akinsegun Akinbami | [segun.akinbami@lasucom.edu.ng](mailto:segun.akinbami@lasucom.edu.ng) |
| NIMR | Dr. Agatha David | nkiru_d@yahoo.com |
| JUTH | Prof. Oche Agbaji | [oagbaji@yahoo.com](mailto:oagbaji@yahoo.com) |
| OOUTH (Sagamu) | Dr. Folashade Adekanmbi | [wonlash@yahoo.com](mailto:wonlash@yahoo.com) |

The PIs at each of the six study sites will oversee all study activities.

### Safety Oversight

All study personnel and Peer Navigators will be trained regarding privacy and confidentiality. They will err on the side of caution and contact the clinical supervisor or site investigator and, if appropriate, escort participants to the nearest emergency room or clinic in the event of an emergency. Both study staff and Peer Navigators will report concerns about safety directly to the PI at their respective site. If any study staff discovers any untreated condition (e.g., onset of physical or mental health condition), they will refer the participant to appropriate treatment immediately. The MPIs (Profs. Garofalo and Taiwo) will be informed of any significant safety concerns within 24 hours of the site PIs becoming aware of the event.

Participant safety will be discussed on weekly team calls, and a written log of all safety events will be maintained, including AEs, SAEs and Unanticipated Problems. Safety will also be monitored through weekly review of peer navigation activity logs, participant encounter forms and monthly tallies of completed satisfaction questionnaires.

AEs and SAEs will be classified by the site PI as related or not related to the study and will be reported to the study team. All serious AEs that are unanticipated will be reported to the IRB, NIH Program Officer, and the DSMB within 3 working days of the study team becoming aware of its occurrence. In consultation with the IRB, the NIH Program Officer, and the DSMB, the study team will notify participants and/or implement modifications to the study protocol.

### Clinical Monitoring

Clinical monitoring will occur according to the local standard of care.

### Quality Assurance and Quality Control

To ensure accuracy of collected data, a monthly data audit will be conducted.

### Data Handling and Record Keeping

#### Data Collection and Management Responsibilities

We have developed systematic protocols for data handling and storage over multiple cohort studies. We maintain both paper files and computer files for each participant. Paper files include: 1) locator information, 2) informed consent, 3) any paper data forms. The first two have identifying information and are linked to the data by the patient identification number (PID).

Computer files consist of the tracking data base, and study data files. Tracking files are maintained in REDCap at Northwestern University, a highly secure web-based research database. This database is used to schedule and track study visits; it is completely password protected. Computer data files never have any identifying information and are encrypted for transfer between study sites. Data files do not include information that could be used to identify the participant from the data file alone.

Dimagi will provide a private cloud server for the SMS application content and user data for the duration of the application deployment. This is a secure system and will be further protected by login credentials for limited access, to protect participant confidentiality. To protect privacy and confidentiality, we will encourage participants to delete text messages after taking medication, to use confidential messages that do not reveal HIV status or mention medications, and we will encourage each participant to protect cell phone confidentiality with a passcode.

#### Study Records Retention

Data files are exported from REDCap and imported into SPSS database for storage and analysis.

### Protocol Deviations

Deviations from this protocol will be reported to the study MPIs and local IRBs as required by local practices.

### Publication and Data Sharing Policy

This protocol will adhere to publications, and data and resource sharing policies of the PATC3H consortium and will be consistent with the relevant NIH policies, laws and regulations.

### Conflict of Interest Policy

Study investigators at IDI/CoMUI, LUTH, NIMR, LASUTH, OOUTH Sagamu, JUTH, and Northwestern University will adhere to their institutional policies for reporting conflicts of interest.

## Additional Considerations

N/A

## Abbreviations and Special Terms

As described in the protocol.

## Protocol Amendment History

*A* ***Summary of Changes*** *table for the current amendment is located in the* ***Protocol Title Page****.*

| **Version** | **Date** | **Description of Change** | **Brief Rationale** |
| --- | --- | --- | --- |
| 2.0 | 22 March 2021 | 1. The study timelines associated with Study 1 and Study 2 were corrected to be consistent with the Study Schema (i.e., Time 1=0-24 weeks)  2. In Study 2, the inclusion criterion for language ability was revised to include two additional languages: Hausa and Pidgin English | 1. The study timelines are now consistent with the Study Schema.  2. In Study 2, the inclusion criterion for language ability now includes the primary languages reflected in the study population. |
| 3.0 | 07 June 2021 | 1. We have removed the enrollment cap of only 35% suppressed participants at clinic sites.  2. The inclusion criteria were revised to allow participation of non-emancipated 15-year-old youth, with parental consent.  3. Exclusion criteria were revised to exclude non-emancipated 15-year-old youth who do not have parental consent.  4. We have removed a reference to DNA extraction in the description of DBS procedures.  5. We have added a requirement for parental consent to the consent procedures.  6.We have revised the consent process for participants who are assented at enrollment, to consent them as the earliest opportunity after they reach the age of consent. | 1. The proportion of suppressed patients varies by clinic site and thus there is no one proportion suppressed that is representative for all clinics.  2. We have broadened the inclusion criteria to include youth who have reached an age of transition to adult care at age 15, but who are not emancipated.  3. Exclusion criteria were revised to make it clear that non-emancipated 15-year-old youth who do not have parental consent are excluded.  4. The reference to DNA extraction in the description of DBS procedures was an error.  5. The inclusion of non-emancipated 15-year old youth in clinical research in Nigeria requires parent consent.  6. We will consent patients who were assented at enrollment to promote their autonomy to consent for research participation. |
| 4.0 | 01 March 2022 | 1. We have updated the description of sample size in study 2 to N=560.  2. In study 1, we have revised the protocol to include abstraction of HIV testing, behavioral and demographic data from sexual partners of eligible individuals tested via iCARE, regardless of sex and age (see updated Consent Statement, Version 2, March 11, 2022).  3. We have updated reporting of SAEs such that only SAEs that are unanticipated, and related or possibly related to the study will be reported to the IRB and the DSMB within 7 working days of the study team becoming aware of its occurrence. All SAEs (regardless of whether they are unanticipated or related or possibly related to the study) will be reported to the Northwestern team and the NIH Program Officer within 3 days of occurrence.  4. With Study PI approval, a remote verbal consent addendum was added to allow HIV treatment arm participants who have moved or are otherwise not available for an in-person follow up study visit at their study site to complete the viral load testing and questionnaires from their current location (see Verbal Consent Addendum, Version 1, March 11, 2022). | 1. The study sample size has been updated for specificity and to align with the Study Schema  2. HIV testing data from sexual partners of eligible cases provides an opportunity to collect additional information on HIV related risk for the target population.  3. The SAE reporting criteria were revised to be consistent with reporting requirements at site IRBs, which only require reporting of SAEs that meet these criteria and to respond to the DSMB preference for reporting.  4. Remote completion of a follow-up study visit will be allowed on rare occasions to limit loss-to-follow-up for the primary endpoint (viral load). |
|  |  |  |  |
|  |  |  |  |
|  |  |  |  |

# REFERENCES

REFERENCES

1. UNAIDS: Joint United Nations Programme on HIV/AIDS. *UNAIDS Data 2019.* Geneva, Switzerland: UNAIDS;2019.

2. Medecins Sans Frontieres. *Out of focus: how millions of people in west and central Africa are being left out of the global HIV response.* Brussles, Belgium: Medicins Sans Frontieres;2016.

3. UNAIDS: Joint United Nations Programme on HIV/AIDS. *Ending AIDS: progress towards the 90-90-90 targets.* Geneva, Switzerland: UNAIDS: Jointed United Nations Programme on HIV/AIDS;2017.

4. Federal Ministry of Health of Nigeria. *National guidelines for HIV prevention, treatment and care, 2016.* 2016.

5. Cohen MS, Chen YQ, McCauley M, et al. Prevention of HIV-1 infection with early antiretroviral therapy. *N Engl J Med.* 2011;365(6):493-505.

6. Insight Start Study Group, Lundgren JD, Babiker AG, et al. Initiation of Antiretroviral Therapy in Early Asymptomatic HIV Infection. *N Engl J Med.* 2015;373(9):795-807.

7. Temprano Anrs Study Group, Danel C, Moh R, et al. A Trial of Early Antiretrovirals and Isoniazid Preventive Therapy in Africa. *N Engl J Med.* 2015;373(9):808-822.

8. Auld AF, Agolory SG, Shiraishi RW, et al. Antiretroviral therapy enrollment characteristics and outcomes among HIV-infected adolescents and young adults compared with older adults--seven African countries, 2004-2013. *MMWR Morb Mortal Wkly Rep.* 2014;63(47):1097-1103.

9. DeSilva MB, Merry SP, Fischer PR, Rohrer JE, Isichei CO, Cha SS. Youth, unemployment, and male gender predict mortality in AIDS patients started on HAART in Nigeria. *AIDS Care.* 2009;21(1):70-77.

10. Mark D, Armstrong A, Andrade C, et al. HIV treatment and care services for adolescents: a situational analysis of 218 facilities in 23 sub-Saharan African countries. *J Int AIDS Soc.* 2017;20(Suppl 3):21591.

11. UNAIDS. *Fast Track-Ending the AIDS Epidemic by 2030.* Geneva, Switzerland: Joint United Nations Programme on HIV/AIDS;2014.

12. UNAIDS. *90-90-90 On the right track towards the global target.* Geneva, Switzerland: Joint United Nations Programme on HIV/AIDS;2016.

13. Adetunji AA, Muyibi SA, Imhansoloeva M, et al. Mobile phone use for a social strategy to improve antiretroviral refill experience at a low-resource HIV clinic: patient responses from Nigeria. *AIDS Care.* 2017;29(5):575-578.

14. Lester R, Karanja S. Mobile phones: exceptional tools for HIV/AIDS, health, and crisis management. *Lancet Infect Dis.* 2008;8(12):738-739.

15. Nigerian Communications Commission. Subscriber statistics. <https://www.ncc.gov.ng/statistics-reports/subscriber-data>. Published 2020. Updated September 24, 2020. Accessed.

16. Centers for Disease Control and Prevention. Compendium of evidence-based interventions and best practices for HIV prevention. Medication adherence (MA) chapter Web site. <https://www.cdc.gov/hiv/research/interventionresearch/compendium/ma/index.html>. Accessed September 18, 2020.

17. Garofalo R, Kuhns LM, Hotton A, Johnson A, Muldoon A, Rice D. A Randomized Controlled Trial of Personalized Text Message Reminders to Promote Medication Adherence Among HIV-Positive Adolescents and Young Adults. *AIDS Behav.* 2016;20(5):1049-1059.

18. Adejumo OA, Malee KM, Ryscavage P, Hunter SJ, Taiwo BO. Contemporary issues on the epidemiology and antiretroviral adherence of HIV-infected adolescents in sub-Saharan Africa: a narrative review. *J Int AIDS Soc.* 2015;18:20049.

19. Resnick MD, Catalano RF, Sawyer SM, Viner R, Patton GC. Seizing the opportunities of adolescent health. *Lancet.* 2012;379(9826):1564-1567.

20. Steinberg L, Monahan KC. Age differences in resistance to peer influence. *Dev Psychol.* 2007;43(6):1531-1543.

21. Patton GC, Sawyer SM, Santelli JS, et al. Our future: a Lancet commission on adolescent health and wellbeing. *Lancet.* 2016;387(10036):2423-2478.

22. World Health Organization. *HIV and adolescents: Guidance for HIV testing and counselling and care for adolescents living with HIV.* Geneva, Switzerland2013.

23. Ammon N, Mason S, Corkery JM. Factors impacting antiretroviral therapy adherence among human immunodeficiency virus-positive adolescents in Sub-Saharan Africa: a systematic review. *Public Health.* 2018;157:20-31.

24. Mark D, Hrapcak S, Ameyan W, et al. Peer Support for Adolescents and Young People Living with HIV in sub-Saharan Africa: Emerging Insights and a Methodological Agenda. *Curr HIV/AIDS Rep.* 2019;16(6):467-474.

25. UNAIDS. UNAIDS estimates. <https://www.unaids.org/sites/default/files/media_asset/unaids-data-2018_en.pdf>. Published 2018. Accessed September 18, 2020.

26. Casale M, Carlqvist A, Cluver L. Recent Interventions to Improve Retention in HIV Care and Adherence to Antiretroviral Treatment Among Adolescents and Youth: A Systematic Review. *AIDS Patient Care STDS.* 2019;33(6):237-252.

27. Grimsrud A, Lesosky M, Kalombo C, Bekker LG, Myer L. Implementation and Operational Research: Community-Based Adherence Clubs for the Management of Stable Antiretroviral Therapy Patients in Cape Town, South Africa: A Cohort Study. 2016;71(1):e16-23.

28. Ojwang VO, Penner J, Blat C, Agot K, Bukusi EA, Cohen CR. Loss to follow-up among youth accessing outpatient HIV care and treatment services in Kisumu, Kenya. *AIDS Care.* 2016;28(4):500-507.

29. Mills EJ, Lester R, Thorlund K, et al. Interventions to promote adherence to antiretroviral therapy in Africa: a network meta-analysis. *Lancet HIV.* 2014;1(3):e104-111.

30. Chaiyachati KH, Ogbuoji O, Price M, Suthar AB, Negussie EK, Barnighausen T. Interventions to improve adherence to antiretroviral therapy: a rapid systematic review. *AIDS.* 2014;28 Suppl 2:S187-204.

31. Johnson AK, Kuhns L, Kuti K, et al. Adaption of evidence based approaches to promote HIV testing and treatment engagement among high risk Nigerian youth. . AIDS Impact; 2019; London, England.

32. Steward W, et al. Peer navigation enhances HIV care retention: An RCT in South African primary clinics. Paper presented at: Conference on Retroviruses and Opportunistic Infections (CROI)2017; Seattle, WA.

33. Lippman SA, Shade SB, Sumitani J, et al. Evaluation of short message service and peer navigation to improve engagement in HIV care in South Africa: study protocol for a three-arm cluster randomized controlled trial. *Trials.* 2016;17:68.

34. Steward WT, Sumitani J, Moran ME, et al. Engaging HIV-positive clients in care: acceptability and mechanisms of action of a peer navigation program in South Africa. *AIDS Care.* 2018;30(3):330-337.

35. Linnemayr S, Huang H, Luoto J, et al. Text Messaging for Improving Antiretroviral Therapy Adherence: No Effects After 1 Year in a Randomized Controlled Trial Among Adolescents and Young Adults. *Am J Public Health.* 2017;107(12):1944-1950.

36. Finitsis DJ, Pellowski JA, Huedo-Medina TB, Fox MC, Kalichman SC. Visual analogue scale (VAS) measurement of antiretroviral adherence in people living with HIV (PLWH): a meta-analysis. *J Behav Med.* 2016;39(6):1043-1055.

37. Proctor E, Silmere H, Raghavan R, et al. Outcomes for implementation research: conceptual distinctions, measurement challenges, and research agenda. *Adm Policy Ment Health.* 2011;38(2):65-76.

38. Damschroder LJ, Aron DC, Keith RE, Kirsh SR, Alexander JA, Lowery JC. Fostering implementation of health services research findings into practice: a consolidated framework for advancing implementation science. *Implement Sci.* 2009;4:50.

39. Magidson JF, Joska JA, Myers B, et al. Project Khanya: a randomized, hybrid effectiveness-implementation trial of a peer-delivered behavioral intervention for ART adherence and substance use in Cape Town, South Africa. *Implement Sci Commun.* 2020;1.

40. Larsen DL, Attkisson CC, Hargreaves WA, Nguyen TD. Assessment of client/patient satisfaction: development of a general scale. *Eval Program Plann.* 1979;2(3):197-207.

41. Balfour L, Kowal J, Tasca GA, et al. Development and psychometric validation of the HIV Treatment Knowledge Scale. *AIDS Care.* 2007;19(9):1141-1148.

42. Erlen JA, Cha ES, Kim KH, Caruthers D, Sereika SM. The HIV Medication Taking Self-efficacy Scale: psychometric evaluation. *J Adv Nurs.* 2010;66(11):2560-2572.

43. Wright K, Naar-King S, Lam P, Templin T, Frey M. Stigma scale revised: reliability and validity of a brief measure of stigma for HIV+ youth. *J Adolesc Health.* 2007;40(1):96-98.

44. Richardson LP, McCauley E, Grossman DC, et al. Evaluation of the Patient Health Questionnaire-9 Item for detecting major depression among adolescents. *Pediatrics.* 2010;126(6):1117-1123.

45. Humeniuk R. *Validation of the alcohol, smoking and substance involvement screening test (ASSIST) and pilot brief intervention: A technical report of phase II findings of the WHO ASSIST project.* Geneva, Switzerland: World Health Organization;2006.

46. *Dedoose* [computer program]. Version 8.0.35. Los Angeles, CA: SocioCultural Research Consultants, LLC; 2018.
